# Simultaneous adrenal and retroperitoneal myelolipoma resected by laparoscopic surgery: a challenging case

**DOI:** 10.1186/s12894-023-01288-5

**Published:** 2023-07-08

**Authors:** Hooman Kamran, Abdolreza Haghpanah, Mohammad Hossein Dabbaghmanesh, Lorenzo Defidio, Maryam Bazrafkan, Anahita Dehghani, Mehdi Shirazi, Nima Naderi, Amirreza Dehghanian

**Affiliations:** 1 Student Research Committee, Shiraz University of Medical Sciences, Shiraz, Iran; 2 Laparoscopy Research Center, Shiraz University of Medical Sciences, Shiraz, Iran; 3 Endourology Ward, Department of Urology, Shiraz University of Medical Sciences, Shiraz, Iran; 4 Department of Urology, Shiraz University of Medical Sciences, Shiraz, Iran; 5 Endocrinology and Metabolism Research Center, Shiraz University of Medical Sciences, Shiraz, Iran; 6 Clinica Nuova Claudia, Rome, Italy; 7 Anesthesiology and Intensive Care Research Center, Shiraz University of Medical Sciences, Shiraz, Iran; 8 Department of Pathology, Shiraz University of Medical Sciences, Shiraz, Iran; 9 Department of Urology, Faghihi Hospital, Zand Avenue, Shiraz, 71348-44119 Iran

**Keywords:** Adrenal glands, Laparoscopy, Myelolipoma, Neoplasms, Retroperitoneal space

## Abstract

**Background:**

Myelolipoma is a benign neoplasm of the adrenal cortex, composed of fat and hematopoietic cells. Although myelolipoma is benign, differentiation from adrenocortical cancer may be difficult. The presence of adrenal and extra-adrenal myelolipomas simultaneously is sporadic, making it a challenging case, especially when the preoperative diagnosis is ambiguous.

**Case presentation:**

A 65-year-old man was referred to our clinic due to a mass in the adrenal fossa. In the abdominopelvic computed tomography (CT), a well-circumscribed fat-containing 78 × 61 × 65 mm bi-lobulated mass was reported in the left adrenal fossa. The first differential diagnosis was myelolipoma. The patient was then referred to our clinic for a mass excision. He was asymptomatic and was scheduled to undergo laparoscopic-assisted adrenalectomy. After adrenalectomy and mass dissection, surprisingly, another mass was detected in the retroperitoneal area. The second mass was also dissected. The final diagnosis was myelolipoma for both masses. The patient has been symptom-free for nine months after the operation.

**Conclusion:**

Simultaneous adrenal and extra-adrenal myelolipoma should be considered as one of the differential diagnoses. However, because this situation is extremely rare, the probability of malignancy should be highly regarded, and we suggest an obsessive approach when approaching this condition. It is essential to manage these cases on a case-by-case basis and tailor the management concerning intraoperative biopsy, the intraoperative appearance of tumors, and the location of extra-adrenal masses.

## Background

Myelolipoma is a benign neoplasm of the adrenal cortex composed of fat and hematopoietic cells [1, 2]. Most myelolipomas are found incidentally due to their asymptomatic presentation [3]. Besides, because of the increase in using abdominal imaging, the incidence rate of these neoplasms is rising, accounting for 6–16% of adrenal incidentalomas [4]. Based on the study by Calissendorff et al. [5], reviewing the epidemiology of adrenal myelolipomas, these masses comprised 3.3–3.6% and 1.8–6.5% of all adrenal tumors in a population and in endocrine clinics, respectively. Also, the prevalence of myelolipoma in the normal population was reported to be 0.32% (at 40 years of age) [6]. In addition, they usually present unilaterally (95%) at the time of diagnosis [5].

Myelolipomatous lesions have three patterns on imaging; isolated adrenal myelolipoma, myelolipoma with hemorrhage, and myelolipomatous foci with other adrenal pathologic conditions [3, 7]. Although myelolipoma is benign, differentiation from adrenocortical cancer may be challenging due to variable fat and hematopoietic portions [1]. Extra-adrenal myelolipoma has been infrequently presented in the literature [8], and retroperitoneal space is the most common location for extra-adrenal myelolipomas [9]. However, myelolipomas in other regions, including the mediastinum and other abdominal organs, have also been reported [10–12]. Based on our search in PubMed, about 130 cases of extra-adrenal myelolipoma have been reported in the English literature. The presence of adrenal and extra-adrenal myelolipomas simultaneously is sporadic, making it a challenging case, especially when the preoperative diagnosis is ambiguous [13]. Here, we aim to present a patient with simultaneous adrenal and retroperitoneal myelolipomas excised by laparoscopic surgery.

### Case presentation

A 65-year-old man with a past medical history of papillary thyroid carcinoma and eye cataract was referred to our clinic due to a mass in the adrenal fossa. On admission, the patient had an approximate body mass index (BMI) of 30 kg/m^2^, the vital signs were stable, and except for the absence of the thyroid gland, the physical exam did not show any abnormalities.

### History

**Fig. 1:**
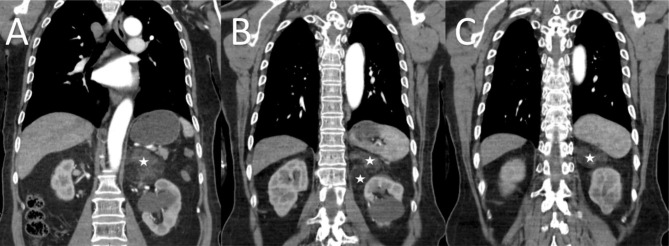
Coronal section of preoperative computed tomography (CT) scan; (**A**) anterior mass, (**B**) both masses, (**C**) posterior mass

The patient underwent radical thyroidectomy due to papillary thyroid carcinoma and took levothyroxine. He had been under follow-up with an endocrinologist regarding this issue. On one of his visits to the endocrinologist, calcium, phosphorous, and hormonal assessment alongside neck, chest and mediastinum, and abdominopelvic computed tomography (CT) scan was ordered to be performed. In the blood test, calcium, phosphorous, T4, antithyroglobulin antibody, and thyroglobulin were within the normal range, but thyroid-stimulating hormone (TSH) was 0.05 (reference range: 0.27–5.95). Also, 24-hour urine levels of cortisol, vanillylmandelic acid (VMA), metanephrine, and normetanephrine, measured to exclude the presence of a possible pheochromocytoma, were within the normal range. However, in the abdominopelvic CT, a well-circumscribed fat-containing 78 × 61 × 65 mm bi-lobulated mass was detected in the left adrenal fossa, resulting in inferior and posterior displacement of the left kidney. The first differential diagnosis was myelolipoma. Besides, no enhancing nodule or solid part was seen (Fig. 1). The patient was then referred to our clinic for a mass excision. He was asymptomatic and was scheduled to undergo laparoscopic-assisted adrenalectomy.

### Hospital course

The patient was admitted to the hospital two days before the operation. Preoperative consultations, including endocrinology, cardiology, and anesthesiology consults, were done, which showed no contraindications for surgery.

**Fig. 2:**
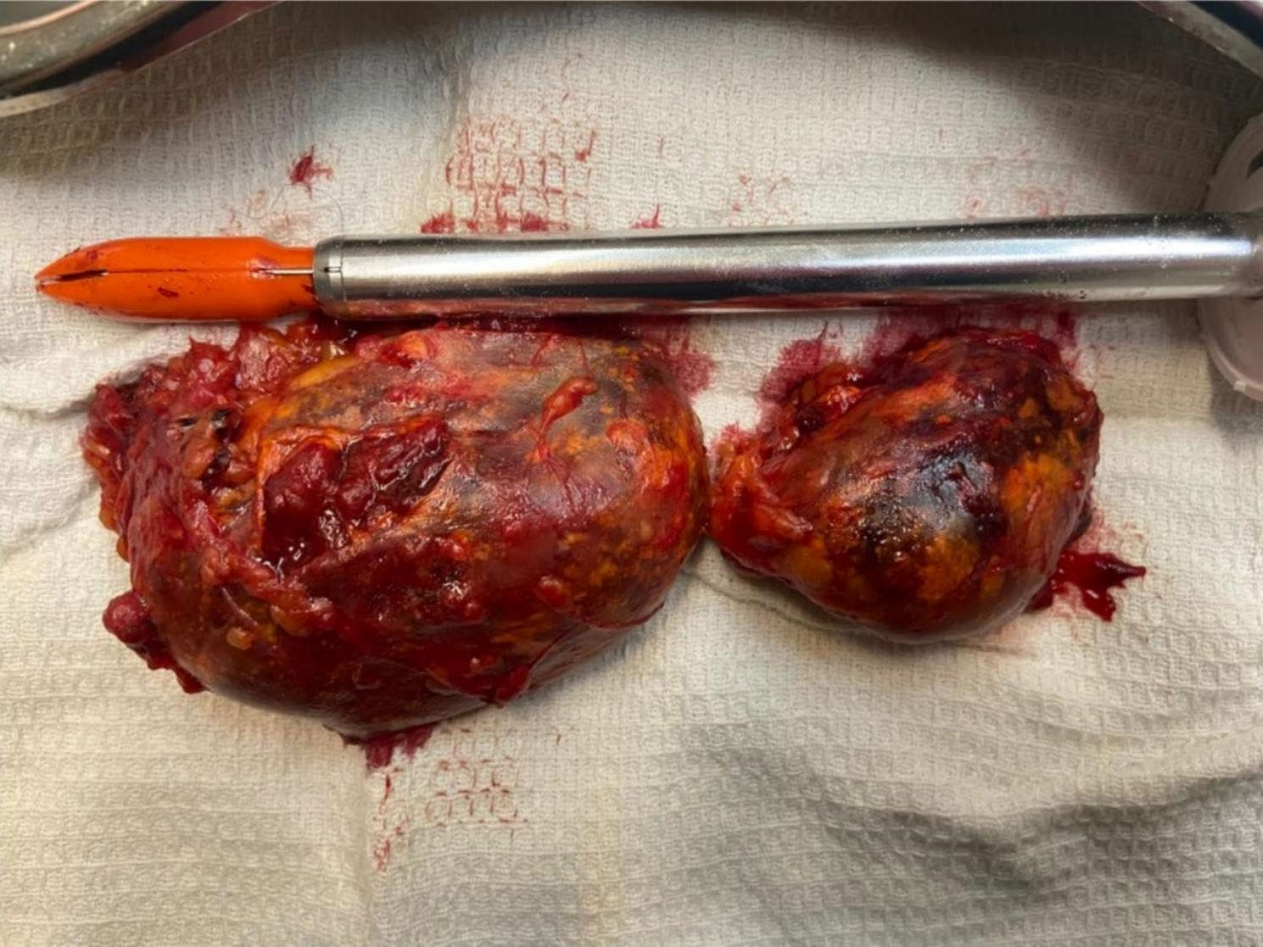
The adrenal and retroperitoneal myelolipomas excised by laparoscopic surgery in a 65-year-old patient

The operation was performed under general anesthesia. The patient was placed in a lateral position. Four ports were inserted, including one for the laparoscope and three for the working ports. After transperitoneal exploration, adrenalectomy was done, and the mass was dissected. Then, when exploring the adrenal fossa, surprisingly, another mass was detected in the retroperitoneal area. The second mass was also dissected, and both masses were excised via a Pfannenstiel incision (Fig. 2). A Penrose drain was inserted, and the incision and trocar sites were closed layer by layer. The operative duration was approximately four hours. The masses were also sent for pathological evaluation.

The postoperative hospital course was uneventful, and the patient was discharged two days after surgery in good condition.

### Pathological evaluation

**Fig. 3:**
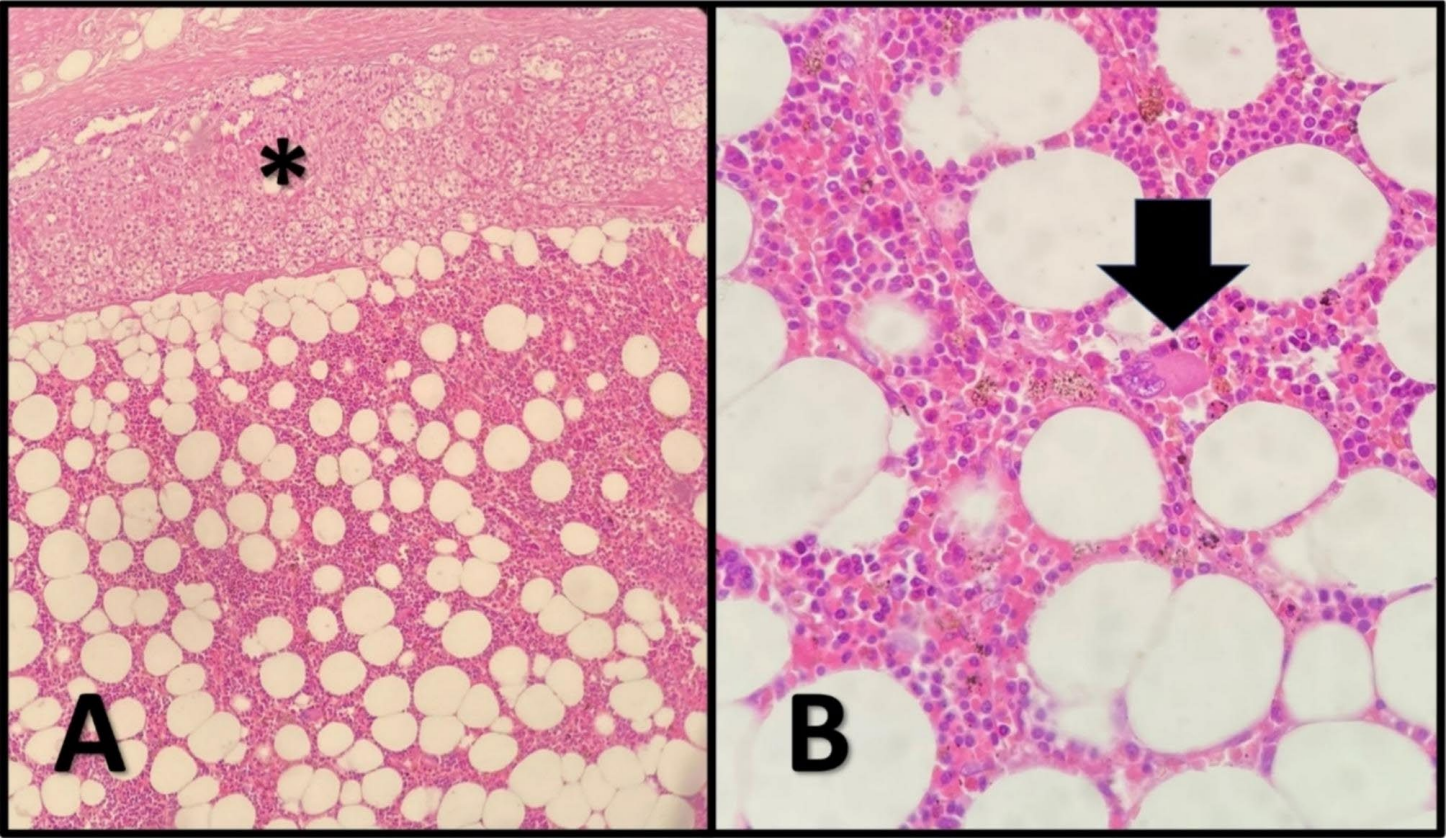
Histopathology of the mass; histopathological sections show normal adrenal cortex (*) just attached to the lesion. The lesion shows two microscopic components consisting of adipose tissue and high cellular patches of hematopoietic cells including megakaryocytes (arrow) justified the diagnosis of myelolipoma of the adrenal (H&E stain, x100, x400)

In the gross examination, both masses were reported as ill-defined masses with creamy yellow color. The size of the adrenal and retroperitoneal masses was 3.5 × 2 × 1 cm and 4 × 3 × 3 cm, respectively. The final diagnosis was myelolipoma for both masses (Fig. 3).

### Follow-up

Currently, the patient is under the follow-up of his endocrinologist. Besides, he has no complaints and has been symptom-free since the operation (a nine-month follow-up).

## Discussion

In the literature, simultaneous adrenal and extra-adrenal myelolipomas are reported exceptionally rarely. The importance of such cases is their challenging management due to the rarity of these situations and the ambiguity of pathologies in the pre-surgical diagnosis. A case report by Zieker et al. [10] presented a 75-year-old man with adrenal and infrarenal myelolipomas. The preoperative diagnosis was a benign adrenal mass alongside a retroperitoneal liposarcoma in their report. So, the authors planned to perform an adrenalectomy with an intraoperative biopsy of the infrarenal mass. Their case shows that the rarity of this presentation and an insecure preoperative diagnosis may challenge the physicians when confronting these patients, especially when the probability of misdiagnosis with malignant tumors is considered. Here, we present a challenging case with simultaneous adrenal and retroperitoneal myelolipomas. In our case, although the preoperative imaging favored myelolipoma, the tumors were reported to be a single bi-lobulated mass. This was while two separated tumors were found during the operation.

The clinical diagnosis of myelolipomas is impractical because most myelolipomas are asymptomatic and are found incidentally. Based on the study by Hamidi et al. [4], which evaluated 321 myelolipomas in 305 patients, 86% of tumors were found incidentally, and only 5% were discovered due to mass effect symptoms. On the other hand, in a review article reviewing 420 cases of adrenal myelolipoma from 1957 to 2017, the most common leading complaints were abdominal discomfort/pain (22.5%) followed by hypochondrial pain (13.9%) and flank pain (13.9%) [1]. Adrenal myelolipomas can rarely present with dyspnea, back pain, fever, weight loss, and virilization [14]. Therefore, myelolipomas-related symptoms, which can result from mass effect symptoms or even acute abdomen because of tumor compression or hemorrhage, are unspecific for diagnosing these tumors [15]. So, further diagnostic approaches are required.

Imaging tools and biopsy are helpful tools for the diagnosis of myelolipomas. In ultrasonography, as the primary imaging tool, myelolipomas have variable appearances based on their composition. Although ultrasound is limited for diagnosis, especially when the mass is small, myelolipomas typically appear as a heterogeneous hyperechoic mass [1, 16]. However, based on the predominant part, it may be hyperechoic (mostly fat) or hypoechoic (mostly myeloid cells) [17]. Also, calcification may be seen [1, 17]. On CT, myelolipomas typically present as well-circumscribed, heterogenous, and hypodense masses, and the presence of fat favor adrenal myelolipoma [1, 16]. However, extra-adrenal myelolipomas have less fat composition and cannot be differentiated clearly from other fat-containing retroperitoneal masses [7, 16]. Also, in magnetic resonance imaging (MRI), hyperintensity of fat on T1 and T2 weighted sequences favor myelolipoma [16]. When imaging cannot exclude malignancy, a fine needle biopsy may be helpful [15].

The differential diagnoses of fat-containing retroperitoneal masses include tumors originating from adrenal (adenoma, myelolipoma), kidney (angiomyolipoma), and pancreas (lipoma, focal pancreatic steatosis), and primary retroperitoneal masses (lipoma, liposarcoma) [18]. Of note, although liposarcoma has been reported to be extremely rare in the adrenal [19, 20], it is one of the most common retroperitoneal fat-containing tumors [16]; the incidence of retroperitoneal liposarcoma has reported to be 2.5 per million population, comprising 20% of all retroperitoneal sarcomas [21]. So, myelolipomas, especially retroperitoneal, may be misdiagnosed as other tumors, including liposarcoma.

Concerning the management of myelolipomas, surgery is indicated in the presence of tumor-associated symptoms with a large-sized mass, suspected malignancy, and tumor growth or hemorrhage [4]. However, conservative management seems sufficient when the diagnosis of myelolipoma is unambiguous and the issues above are absent. As a benign tumor, laparoscopic surgery is safe, and follow-up is optional because the malignant transformation has not been reported [1, 15].

**Fig. 4:**
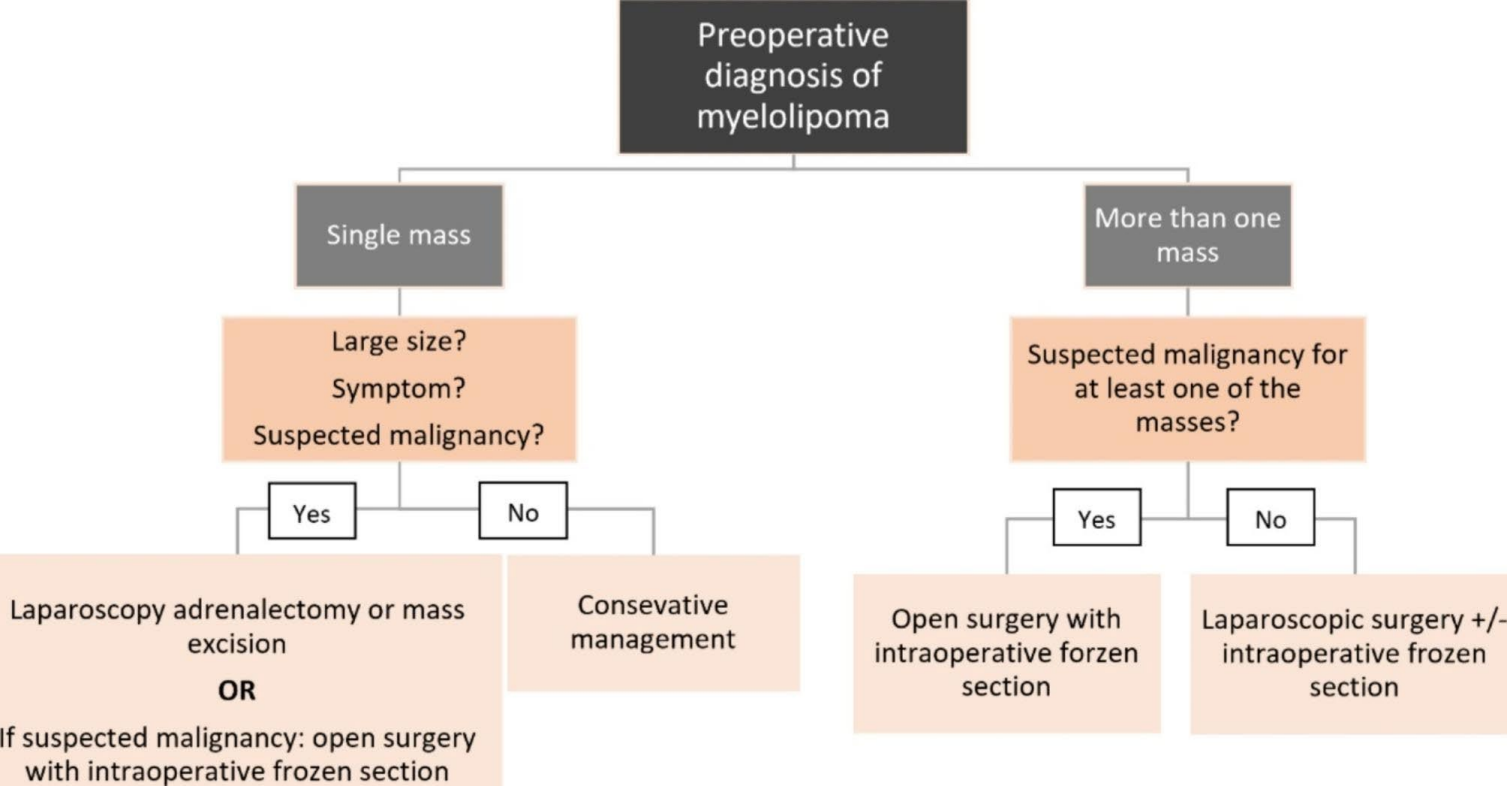
Simplified algorithm for the management of tumors with preoperative diagnosis of myelolipoma

The management of simultaneous adrenal and extra-adrenal myelolipoma is more challenging. One reason is the extreme rarity of these cases. So, the management must be considered on a case-by-case basis. Our report and the report by Zieker et al. [13] show that simultaneous presentation of myelolipoma should be considered when it is suspicious in the pre-treatment imaging. However, we suggest a more obsessive approach when confronting these patients. Because this situation is extremely rare, the probability of malignancy, especially retroperitoneal liposarcoma, should be highly considered. We designed a simplified algorithm when preoperative single or multiple myelolipomas are considered (Fig. 4). According to the algorithm, open surgery with intraoperative biopsy is suggested when malignancy is suspected in multiple myelolipomas. On the other hand, laparoscopic surgery is indicated when malignancy is not suspected; however, due to the reasons above, a more obsessive approach is recommended by performing an intraoperative biopsy. Although this algorithm may help manage such cases, it is simplified, and managing these challenging situations must be made case by case. This algorithm is just a simplified approach showing our opinion on the subject and is not a validated algorithm. The management should be tailored regarding intraoperative biopsy, the intraoperative appearance of tumors, and the location of extra-adrenal masses.

In our case, because the mass was preoperatively considered to be a single bi-lobulated myelolipoma, we scheduled a laparoscopic surgery due to the large size of the mass and the bi-lobulated appearance of the mass, considering the probability of separate masses. And surprisingly, two masses were seen during the operation. After adrenalectomy, the adrenal mass was observed to be smaller than the reported size in the preoperative CT scan. So, the retroperitoneal space was explored, and a retroperitoneal mass was found. Based on the lack of suspected malignancy and the appearance of the retroperitoneal mass, which was round, well-differentiated, and utterly similar to the excised adrenal mass, no intraoperative frozen section was sent. In the case by Zieker et al. [13], the management was done similar to our algorithm, and open surgery with intraoperative biopsy was performed due to the suspected malignancy for the retroperitoneal mass.

## Conclusion

As the presence of simultaneous adrenal and extra-adrenal myelolipoma is extremely rare, its management is more challenging than a single mass. It should be considered as one of the differential diagnoses. However, because this situation is extremely rare, the probability of malignancy, especially retroperitoneal liposarcoma, should be highly considered, and we suggest an obsessive approach when approaching this condition. It is important to manage these cases on a case-by-case basis and tailor the management concerning intraoperative biopsy, the intraoperative appearance of tumors, and the location of extra-adrenal masses. Of note, as this study is only a case report, maybe, the proposed algorithm cannot be generalized to all patients presenting with simultaneous adrenal and extra-adrenal myelolipoma in the future. Therefore, future reports may help the literature on the approach to simultaneous adrenal and extra-adrenal tumors, including myelolipomas.

## Data Availability

All information of the patients is presented in the manuscript.
